# Effects of incentive-based population policies on sustainability of China’s recent maternity insurance system reform: a system dynamics simulation

**DOI:** 10.1186/s12961-022-00945-9

**Published:** 2022-12-28

**Authors:** Xiaotian Zhang, Xiaoyun Liu, Wanxin Wang, Lulin Zhou, Yang Wang, Mingyue Li

**Affiliations:** 1 China Centre for Health Development Studies, Peking University, Beijing, 100191 People’s Republic of China; 2 Tunbridge Wells Hospital, Tonbridge Rd, Royal Tunbridge Wells, TN2 4QJ United Kingdom; 3 School of Management, Hospital Management and Health Policy Research, Centre for Medical Insurance, Jiangsu University, Jiangsu, People’s Republic of China

**Keywords:** Fertility rate, Grounded theory, System dynamics model, Economic incentives, Risk resistance, Sustainable development

## Abstract

**Background:**

This paper seeks to assess the sustainability of the reformed maternity insurance system and the extent to which China’s current maternity insurance system can support different levels of fertility incentives in the future. Our findings will serve as a reference for countries in a similar demographic predicament and those about to face it.

**Methods:**

This study used a combination of qualitative and quantitative methods. In the qualitative assessment, we used a grounded theory model to generalize the factors influencing the sustainability of maternity insurance funds. For the quantitative analysis, we used a novel and comprehensive system dynamics model to visualize the status of the combined operation of maternity and health insurance. Data are mainly derived from the historical data of the Statistical Yearbook of Jiangsu Province and the National Bureau of Statistics of China.

**Results:**

In the short term, fertility incentive payments can be set to motivate people to have children. It is therefore recommended that when the scope of the fertility incentive policy is limited to two children, and an average amount above RMB 10 000 could be set, it would be prudent to set the amount at a level not exceeding RMB 10 000 when the scope of the fertility incentive policy is for all newborns. In the long term, a system of incentives for childbirth should be built from education policy, house price regulation, tax relief and childcare services.

**Conclusion:**

Our research not only highlights the significance of improving the resilience of maternity insurance by combining maternity insurance and health insurance funds, but also suggests a way to economically incentivize beneficiaries to have children so as to mitigate the decline in China’s birth rate and cope with the crisis of an ageing population.

## Background

The number of births in China has been rapidly declining. A drop of 14% in the number of newborns has been observed, from 17.23 million in 2017 to 14.65 million in 2019 [1]. China abolished the existing one-child population and family planning policy in 2015. This was replaced with a comprehensive two-child policy [2]. Although the comprehensive two-child policy has been effective in increasing the number of births, the actual population growth has remained lower than expected [3]. Therefore, formulating strategies to address people’s willingness to conceive and to increase the fertility rate has become an urgent issue for China.

China established a maternity insurance scheme in 1964 to ensure a high maternal survival rate. According to a recent study [4], the current comprehensive two-child policy in China has already led to unsustainability of the maternity insurance fund, and this unsustainability will be further exacerbated if a maternity incentive policy is introduced without any change to the existing payment rates. China is planning to merge national health insurance with maternity insurance. These reforms aim to increase the risk resilience of the maternity insurance fund. China started a pilot reform to merge national health insurance with maternity insurance in individual cities in 2018 [5]. However, this policy has not been scaled up to the whole country so far.

Since declining birth rates have become a social concern for many countries after a certain level of economic development, the development of a reasonable and scientific policy on fertility incentives is strategically important. The significant impact of economic incentives on fertility promotion is now internationally recognized. Laroque et al. [6] found that financial incentives have had a significant effect on fertility decisions in France. The province of Quebec in Canada introduced a pronatalist transfer policy to pay up to Can$ 8 000 to families having a child. Research found that it had a strong effect on fertility rates [7]. Tudor found that a change in financial incentives did not influence short-term conception rates but significantly decreased the probability of abortions, leading to more live births; thus, the change in financial incentives increased fertility in the long term [8]. Yasuoka [9] studied the Japanese policy and concluded that child allowance has a positive effect on increasing fertility. Langridge et al. [10] examined the Australian government’s introduction of a maternity allowance policy known as the baby bonus and found that for countries with social, economic and political climates similar to Australia, a monetary incentive may provide a satisfactory solution to declining general fertility rates. Ang [11] found that paid parental leave is a cost-effective way to increase fertility, whereas the price per additional birth due to cash-transfer fertility incentives is quite high.

Internationally, several scholars have studied social insurance from the perspective of the sustainability of various types of social insurance funds. Nyandekwe et al. studied Rwandan community-based health insurance and found that financial sustainability was achievable, but it was contingent upon the persistence of political commitment efforts to achieve universal health coverage, correction of highlighted imperfections and injection of additional funding [12]. Yao et al. proposed a risk-adjusted subsidy provided by the government to micro-insurers as a method to enhance micro-health insurance for maternity benefits in Pakistan. They posited that this payment model could improve efficiency and sustainability by extending affordable maternity care to low-income women in developing regions [13]. Habicht et al. [14] found that the Estonian government addressed the long-standing challenge of financial sustainability of the health system by expanding its revenue base [12].

Many other scholars have studied the sustainability of various types of social insurance in China, such as China’s urban workers’ medical insurance fund (UWMIF) [15–18], urban residents’ medical insurance fund (URMIF) [19], pension insurance fund (PIF) [20], new rural cooperative fund (NRCF) [21] and work injury insurance fund (WIIF) [22]. Chen [23] found that the sustainability of social insurance systems, including maternity insurance systems and medical insurance systems, is in fact the sustainability of social insurance funds. Zhan et al. [24] found that it is necessary to set a reasonable contribution rate for the maternity insurance fund based on people’s demand for a maternity insurance system. Some experts have also argued that the balance between funding and payment is the life force of a sustainable social insurance system, and that the fund balance is the top priority for the sustainable development of a maternity insurance system [25].

At present, there are few studies on the sustainable development of maternity insurance systems, and they mainly focus on the impact of population policies on the maternity insurance system [2, 4]. Some researchers have focused on specific maternity insurance policies, for example, increasing the maternity allowance, operation of the maternity insurance fund and reform of the maternity insurance system [26–29].

Most previous research has focused on cost control. Research on the factors influencing the sustainable development of maternity insurance funds is relatively scarce. Few studies use qualitative research tools to systematically investigate the implementation of the maternity insurance fund. Current methods mostly involve using actuarial models, and there is no intuitive system dynamics model to describe the sustainable development of a maternity insurance fund, let alone a system that is integrated with the possible future maternity incentive policies in China.

China has not yet introduced a comprehensive and systematic policy to encourage childbirth. Whatever type of maternity incentives are introduced, the maternity insurance system should play a supportive role. In this context, in the future, after the merger of maternity insurance and medical insurance, the ability of the insurance fund to support the maternity incentive policy and its own sustainability is a question worth examining. This paper seeks to assess the sustainability of the reformed maternity insurance system and the extent to which China’s current maternity insurance system can support different levels of fertility incentives in the future. Our findings will serve as a reference for countries in a similar demographic predicament and those about to face it.

## Methods

### Study design

This study used a combination of qualitative and quantitative methods. The qualitative analysis draws on the findings of earlier work by the authors. We use four multi-stakeholder workshops and in-depth interviews to bring together three groups of people—maternity insurance system developers, implementers and researchers. We then analyse the factors influencing the sustainability of the maternity insurance system using grounded theory [30].

The quantitative section builds on the findings of the qualitative study to construct a system dynamics model of the sustainability of the maternity insurance system. The realistic data were then brought in and simulated for the sustainability of the reformed maternity insurance system, as well as for predicting the extent to which China’s current maternity insurance system will be able to support different levels of maternity incentives in the future.

### Data source

For the qualitative analysis, in order to ensure the reliability and validity of research data, this study used the purposive sampling method to collect relevant information from multiple authoritative sources. These mainly include professors and scholars of social security research from some top universities in Jiangsu Province; relevant experts from Jiangsu Provincial Medical Insurance Bureau, Jiangsu Provincial Human Resources and Social Security Bureau and Jiangsu Provincial Medical Insurance Settlement Centre; and related experts and policy implementers of human resources and social security bureaus and health insurance bureaus in various cities of Jiangsu Province. In addition, relevant texts and public media materials were collected. Workshops and in-depth interviews are commonly used for qualitative research, and the interactive process helps researchers brainstorm and dig deeper into their concerns.

In the quantitative analysis, data were procured from the statistical yearbooks of Jiangsu Province and the National Bureau of Statistics of China. These include data about the population, birth rate and death rate of Jiangsu Province, and the fund balance, number of insured individuals, fund income, fund expenditure, wage of insured persons and number of beneficiaries of maternity and health insurance. Data were collected for a period of 14 years, from 2006 to 2019.

Jiangsu Province is one of the most economically prosperous provinces in China, and one of the first provinces in China to implement pilot maternity insurance system reforms. This article is a series of studies based on the results of previous research combined with the latest available data [4].

### Grounded theory model

Conceptualization of the data collected using a three-level coding approach begins with an initial code. Analysis of the initial coding then allows for the generalization of conceptual categories, and theoretical coding ultimately leads to a model of the factors influencing the sustainability of maternity insurance funds. This paper uses NVivo 12.0 and Excel software to analyse data in order to investigate factors influencing the sustainability of funds through a grounded theory approach.

**Fig. 1:**
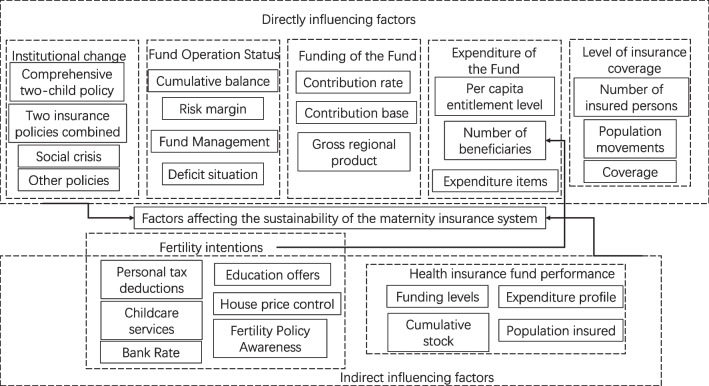
Model of influencing factors for sustainable development of the maternity insurance fund

Through in-depth mining analysis of the seven core categories obtained from axial coding and their corresponding categories, as well as a continuous comparative analysis with the initial open coding and the original data, a theoretical coding of the factors influencing the sustainability of the maternity insurance fund was developed. The "model of influencing factors for sustainable development of the maternity insurance fund" was obtained, as shown in Fig. 1 [30].

### System dynamics model

Grounded theory was applied to distil and summarize the factors influencing the sustainable development of maternity insurance funds, ultimately constructing a theoretical model of the sustainable development of said funds. However, there is a need for more systematic and detailed models, as the theoretical model is limited by difficulties in carrying out simulations with real data, as well as in visualizing the process of sustainable development in the empirical study. Previous research on the sustainability of maternity funds had mostly employed structural equations and actuarial models, which are fine-grained but not sufficiently visual to provide an intuitive view of the entire system. There is a lack of visualization and generalized system models, so this study introduces system dynamics into the mix. Through the systematic integration and analysis of factors within the fund, computer simulations are used to predict the operation of the maternity insurance fund. This will help the government to implement new maternity policies and to calculate optimization strategies to maintain the sustainability of maternity funds by incorporating empirical data.

The latest data from the Jiangsu Provincial Bureau of Statistics as of 2019 showed that the accumulated balance of urban employees’ medical insurance in Jiangsu Province is RMB 182.232 billion, showing a continuous high trend compared to the accumulated balance in previous years. The accumulated balance of maternity insurance is RMB 3.54 billion, which is about 51 times smaller than the former. Implementation of the merger of medical insurance and maternity insurance can very well drive the maternity insurance fund, which is on the verge of deficit, out of its predicament and achieve sustainable development.

According to the results of the qualitative study on the impact of the sustainable development of the maternity insurance fund, the system of merging the two insurance systems involves the main areas of institutional change, operation of the maternity insurance fund, financing of the maternity insurance fund, expenditure of the maternity insurance fund, the degree of maternity insurance coverage, the willingness to give birth, and operation of the medical insurance fund. Specifically, it includes the income of the maternity insurance fund, the expenditure of the maternity insurance fund, the transfer of the maternity insurance fund, the participation rate of the maternity insurance, the contribution rate of the maternity insurance, the income of the urban employees’ medical insurance fund, the expenditure of the urban employees’ medical insurance fund, the participation rate of the urban employees’ medical insurance, the contribution rate of the urban employees’ medical insurance, the number of urban employees’ medical insurance participants, and the financing of the urban employees’ medical insurance. The rate of participation in urban employees’ medical insurance, the rate of contribution to urban employees’ medical insurance, the number of urban employees’ medical insurance participants and the funding standard for urban employees’ medical insurance are also included.

The income of the maternity insurance and medical insurance coordination fund is equal to the sum of the income of the maternity insurance fund and the income of the urban employees’ medical insurance, and the expenditure of the maternity insurance and medical insurance coordination fund is equal to the sum of the expenditure of the maternity insurance fund and the expenditure of the urban employees’ medical insurance. However, as the number of participants in maternity insurance and urban employees’ medical insurance overlap but do not completely coincide, and the contribution bases, methods and rates are different, the two have their own separate demographic and economic subsystems.

#### Total income of the fund after the merger of maternity and health insurance

The financing module of the maternity insurance and medical insurance funds consists of the income from the basic medical insurance for urban workers and the income from the maternity insurance fund, both of which are additive. The income from the basic urban workers’ medical insurance is more complex, comprising individual and unit contributions and government subsidies; the income from the maternity insurance fund is derived by multiplying the base amount of maternity insurance contributions with the rate of maternity insurance contributions.

The formula is summarized as follows:

$$({\text{MFI}})_{t} = ({\text{MPR}})_{t} \times ({\text{MPB}})_{t} = ({\text{MPR}})_{t} \times \left[ {({\text{MAP}})_{t} \times ({\text{MIN}})_{t} } \right],$$

where $({\text{MFI}})_{t}$ is the maternity insurance fund income in year *t*, $({\text{MPR}})_{t}$ is the payment rate of maternity insurance in year *t*, $({\text{MPB}})_{t}$ is the payment base of maternity insurance in year *t*, $({\text{MAP}})_{t}$ is the average wage of people insured by maternity insurance in year *t*, and$({\text{MIN}})_{t}$ is the number of people insured by maternity insurance in year *t*.

Therefore,

$$({\text{TFI}})_{t} = ({\text{MFI}})_{t} + ({\text{MEFI}})_{t},$$

where $({\text{TFI}})_{t}$ denotes the total income of the coordinated fund in year *t* after the merger of maternity insurance and urban employees’ medical insurance, and$({\text{MEFI}})_{t}$ denotes the income of the medical insurance fund for urban employees in year *t*.

#### Total expenditure of the fund after the merger of maternity and health insurance

The expenditure module of the maternity insurance and urban employees’ medical insurance funds consist mainly of the expenditure of the basic medical insurance for urban employees and the expenditure of the maternity insurance fund, and the two are additive. The expenditure of the basic medical insurance for urban workers includes medical expenses and risk reserves; the expenditure of the maternity insurance fund is the multiplication of the per capita level of maternity insurance benefits and the number of beneficiaries. In the subsequent simulation study, a separate maternity incentive payment from the fund will be created and added to the per capita level of maternity insurance benefits.

$$\begin{aligned} ({\text{MFE}})_{t} & = ({\text{MAT}})_{t} \times ({\text{MEN}})_{t} + ({\text{TIP}})_{t} \\ & = ({\text{MAT}})_{t} \times \left\{ {\left[ {\left( {({\text{MIN}})_{t} + {\text{SN}}_{t} } \right) \times {\text{br}}_{t} } \right] - \left[ {\left( {({\text{MIN}})_{t} + {\text{SN}}_{t} } \right) \times {\text{dr}}_{t} } \right]} \right. \\ & \quad - \left. {\left[ {0.011236\left( {({\text{MIN}})_{t} + {\text{SN}}_{t} } \right) \times {\text{br}}_{t} } \right]} \right\} + \left[ {({\text{AIP}})_{t} \times ({\text{nact}})_{t} } \right], \\ \end{aligned}$$

where$({\text{MFE}})_{t}$ is the maternity insurance fund expenditure in year *t*, $({\text{MAT}})_{t}$ is the maternity insurance’s average level of treatment in year *t*, $({\text{MEN}})_{t}$ is the maternity insurance’s number of beneficiaries in year *t*, $({\text{TIP}})_{t}$ is total fertility incentive payments from the maternity insurance funds in year *t*, $({\text{AIP}})_{t}$ is average incentive payments for beneficiaries in year *t*, $({\text{nact}})_{t}$ denotes the number of new second children with maternity insurance in year *t* in Jiangsu Province, ${\text{br}}_{t}$ is the birth rate in year *t*, ${\text{dr}}_{t}$ is the death rate in year *t*, and ${\text{SN}}_{t}$ is the new insured people with maternity insurance in year *t*. The coefficient 0.011236 is the probability of twins.

Therefore,

$$({\text{TFE}})_{t} = ({\text{MFE}})_{t} + ({\text{MEFE}})_{t},$$

where $({\text{TFE}})_{t}$ denotes the total expenditure of the maternity insurance and medical insurance coordination fund in year *t*, and $({\text{MEFE}})_{t}$ denotes the expenditure of the urban employees’ medical insurance fund in year *t*.

#### Number of second children in Jiangsu Province after implementation of the comprehensive two-child policy

According to the Statistical Yearbook 2020 of the Jiangsu Provincial Bureau of Statistics, the number of births in Jiangsu Province in 2015 before the implementation of the comprehensive two-child policy was approximately 721 100. The number of births in Jiangsu Province in 2016 and 2017 after implementation of the comprehensive two-child policy was approximately 779 600 and 778 200, respectively. It is assumed here that the increase of 57 800 is due to the stimulation of the comprehensive two-child policy, but since the number of people insured by maternity insurance in Jiangsu Province is smaller than the number of people in the province, a ratio needs to be multiplied.

The formula is summarized as follows:

$$({\text{nact}})_{t} = ({\text{NCT}})_{t} \times \frac{{({\text{MIN}})_{t} }}{{({\text{TP}})_{t} }},$$

where $({\text{nact}})_{t}$ denotes the number of new second children with maternity insurance in year *t* in Jiangsu Province, $({\text{NCT}})_{t}$ denotes the number of new second children in Jiangsu Province in year *t*, $({\text{MIN}})_{t}$ denotes the number of maternity insurance participants in Jiangsu Province in year *t*, and $({\text{TP}})_{t}$ denotes the total population in Jiangsu Province in year *t*.

According to the 2020 Jiangsu Statistical Yearbook, the number of maternity insurance participants in Jiangsu Province as a percentage of the total population of Jiangsu Province is basically maintained at around 0.18. The calculation shows that the number of new children in Jiangsu Province is about 10 000 per year.

### Visualization of the system dynamics model

**Fig. 2:**
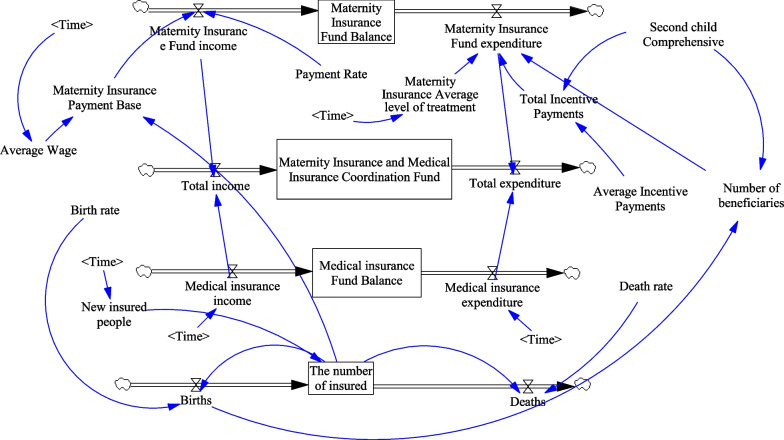
System dynamics diagram for the future implementation of a combined maternity and health insurance system

According to the results of the qualitative study above, the whole coordinated fund system includes the subsystem of the maternity insurance fund, the subsystem of urban employees’ medical insurance fund, the economic subsystem, the population subsystem and the policy subsystem. The maternity insurance fund subsystem includes the accumulated balance of the maternity insurance fund, and the current year’s fund balance, income, expenditure, enterprise contribution rate, average incentive payment and total incentive payment; the urban employees’ medical insurance fund subsystem includes total income and total expenditure; the population subsystem mainly includes the number of insured persons and the number of new children under the two-child policy; and the economic subsystem includes the average annual wage of employed workers. The variables interact with each other to form a net structure. In addition, the economic system includes the maternity insurance transfer fund and the health insurance risk reserve fund, which mainly include investment appreciation and interest. As they are included in the income of both insurance funds and the amount is small, they will be ignored in this study. The final system dynamics flow diagram is shown in Fig. 2.

Due to the fact that the study’s scope of view focuses on the maternity insurance system, the factors of the urban employees’ health insurance system are simplified in the system dynamics flow diagram, and both income and expenditure are subdivided in the form of a fitted function. In this paper, it was found that due to the unavailability of data collection for the refinement, such a fit could instead improve the model accuracy.

### Summary of model formulas

$$\begin{aligned} S\left( {t_{x} } \right) & = S\left( {t_{x - 1} } \right) + \int_{{t_{x - 1} }}^{{t_{x} }} {{\text{rate}}\;S\left( {t_{x} } \right){\text{d}}t} \\ & = S\left( {t_{x - 1} } \right) + \left[ {({\text{TFI}})_{{t_{x} }} - ({\text{TFE}})_{{t_{x} }} } \right] \times 1 = S\left( {t_{x - 1} } \right) + \left[ {({\text{TFI}})_{{t_{x} }} - ({\text{TFE}})_{{t_{x} }} } \right] \\ \end{aligned},$$

$$\begin{aligned} \left( {{\text{MMCF}}} \right)_{{t_{x} }} & = S\left( {t_{0} } \right) + \left[ {\left( {\int_{{t_{0} }}^{{t_{1} }} {{\text{rate}}\;S(t_{1} )} {\text{d}}t} \right) + \cdots + \left( {\int_{{t_{x - 1} }}^{{t_{x} }} {{\text{rate}}\;S(t_{x} ){\text{d}}t} } \right)} \right] \\ & = S\left( {t_{0} } \right) + \sum\limits_{{t = t_{1} }}^{{t_{x} }} {\left( {\int_{{t_{x - 1} }}^{{t_{x} }} {{\text{rate}}\;S\left( {t_{x} } \right){\text{d}}t} } \right)} \\ & = S\left( {t_{0} } \right) + \sum\limits_{{t = t_{1} }}^{{t_{x} }} {\left\{ {\left[ {({\text{TFI}})_{{t_{1} }} - ({\text{TFE}})_{{t_{1} }} } \right] + \left[ {({\text{TFI}})_{{t_{2} }} - ({\text{TFE}})_{{t_{2} }} } \right] + \cdots + \left[ {({\text{TFI}})_{{t_{x} }} - ({\text{TFE}})_{{t_{x} }} } \right]} \right\}} \\ & = S\left( {t_{0} } \right) + \sum\limits_{{t = t_{1} }}^{{t_{x} }} {\left\{ {\left[ {({\text{TFI}})_{t} - ({\text{TFE}})_{t} } \right]} \right\}} \\ \end{aligned},$$

where $({\varvec{MMCF}})_{{t_{x} }}$ is the accumulated balance of the maternity insurance fund after the merger with the urban employees’ medical insurance. The formula $S\left( {t_{x} } \right) = ({\text{MMCF}})_{{t_{x} }}$ denotes the cumulative total balance of the maternity insurance and urban employees’ medical insurance coordination fund, and $S\left( {t_{0} } \right)$ denotes the accumulated balance of the maternity insurance and urban employees’ medical insurance coordinated fund in year *t*_0_.

$$S\left( {t_{0} } \right) = S\left( {t_{0} } \right)^{\prime } + S\left( {t_{0} } \right)^{\prime \prime },$$

where $S\left( {t_{0} } \right)^{\prime }$ denotes the accumulated balance of the maternity insurance fund in year *t*_0_, and $S\left( {t_{0} } \right)^{\prime \prime }$ denotes the accumulated balance of the urban employees’ medical insurance fund in year *t*_0_.

$$({\text{TFI}})_{t} = ({\text{MFI}})_{t} + ({\text{MEFI}})_{t},$$

where $({\text{TFI}})_{t}$ denotes the total income of the maternity insurance and urban employees’ medical insurance coordinated fund in year *t*.

$$({\text{TFE}})_{t} = ({\text{MFE}})_{t} + ({\text{MEFE}})_{t},$$

where $({\text{TFE}})_{t}$ denotes the total expenditure of the maternity insurance and urban employees’ medical insurance coordination fund in year *t*.

Therefore,

$$\begin{aligned} ({\text{MMCF}})_{{t_{x} }} & = S^{\prime\prime}\left( {t_{0} } \right) + \mathop \sum \limits_{{t = t_{1} }}^{{t_{x} }} \left\{ {\left[ {({\text{TFI}})_{t} - ({\text{TFE}})_{t} } \right]} \right\} \\ & = \left[ {S\left( {t_{0} } \right)^{\prime } + S\left( {t_{0} } \right)^{\prime \prime } } \right] + \mathop \sum \limits_{{t = t_{1} }}^{{t_{x} }} \left\{ {\left[ {({\text{MFI}})_{t} + ({\text{MEFI}})_{t} } \right] - \left[ {({\text{MFE}})_{t} + ({\text{MEFE}})_{t} } \right]} \right\} \\ & = \left[ {S\left( {t_{0} } \right)^{\prime } + S\left( {t_{0} } \right)^{\prime \prime } } \right] + \mathop \sum \limits_{{t = t_{1} }}^{{t_{x} }} \left\{ \begin{gathered} \left[ {\left( {\left[ {({\text{MPR}})_{t} \times ({\text{MAP}})_{t} \times ({\text{MIN}})_{t} } \right]} \right) + ({\text{MEFI}})_{t} } \right] \hfill \\ - \left[ \begin{gathered} \left( {\left( {{\text{MAT}}} \right)_{t} \times \left( \begin{gathered} \left( {\left( {({\text{MIN}})_{t} + {\text{SN}}_{t} } \right) \times {\text{br}}_{t} } \right) - \hfill \\ \left( {\left( {({\text{MIN}})_{t} + {\text{SN}}_{t} } \right) \times {\text{dr}}_{t} } \right) - \hfill \\ \left( {0.011236\left( {({\text{MIN}})_{t} + {\text{SN}}_{t} } \right) \times {\text{br}}_{t} } \right) + \hfill \\ \left( {{\text{nact}}} \right)_{t} \hfill \\ \end{gathered} \right) + \left( {{\text{TIP}}} \right)_{t} } \right) \hfill \\ + \left( {{\text{MEFE}}} \right)_{t} \hfill \\ \end{gathered} \right] \hfill \\ \end{gathered} \right\} \\ \end{aligned}.$$

### Model variables and functional relationships

The variables and functional relationships of the system dynamics model for the sustainable development of the future maternity insurance fund after the merger are shown in Table 1, mainly derived from the Jiangsu Provincial Statistical Yearbook and historical data obtained from research.

**Table 1:** Variables and functional relationships in the system dynamics model of the sustainable development of the maternity insurance fund after the merger of the two insurance systems

No.	Variable name	Meaning	Type	Variable expression and assignment	Units
1	Maternity insurance average level of treatment	Average entitlement level of maternity insurance	Auxiliary variables	3 351 × time − 6.7203e+006	RMB
2	Average wage	Average payment base of maternity insurance	Auxiliary variables	3 514 × time − 7.0354e+006	RMB
3	Birth rate	Births	Auxiliary variables	0.00937	Rate
4	Births	Number of births	Rate variables	Number of insured × birth rate	Person/year
5	Death rate	Mortality	Auxiliary variables	0.007	Rate
6	Deaths	Number of deaths	Rate variables	Number of insured × death rate	Person/year
7	Number of beneficiaries	Maternity insurance’s number of beneficiaries	Auxiliary variables	Births − births × 0.011236 + second child comprehensive	Persons
8	Final time	System length	Auxiliary variables	2027	Years
9	Maternity insurance fund balance	Maternity insurance base	Status variables	INTEG (fund income − fund expenditure, 2.761e+009)	RMB/year
10	Maternity insurance fund expenditure	Expenditure of maternity insurance fund	Rate variables	Maternity insurance average level of treatment × number of beneficiaries + total incentive payments	RMB/year
11	Maternity insurance fund income	Income of maternity insurance fund	Rate variables	Payment base × payment rate	RMB/year
12	INITIAL TIME	System start time	Auxiliary variables	2017	Years
13	New insured people	Number of new insured per year of maternity insurance	Auxiliary variables	Assigning values based on different scenarios	Persons
14	Maternity insurance payment base	Payment base of maternity insurance	Auxiliary variables	Average wage × number of insured	RMB
15	Maternity insurance payment rate	Contribution rate of maternity insurance	Auxiliary variables	0.005	Rate
16	Second child comprehensive	Total number of additions per year	Auxiliary variables	Assigning values based on different scenarios	Persons
17	Number of insured	Number of insured persons	Status variables	INTEG (births − deaths + new insured people, 1.51032e+007)	Person/year
18	Maternity insurance and medical insurance coordination fund	Maternity and health insurance consolidated fund	Status variables	INTEG (total income − total expenditure, 1.1394e+011)	RMB
19	Medical insurance fund balance	Accumulated balance of the medical insurance fund	Status variables	INTEG (medical insurance income − medical insurance expenditure, 1.11179e+011)	RMB
20	Medical insurance income	Income volume of the medical insurance fund	Rate variables	7.89833e+009 × time − 1.58369e+013	RMB/year
21	Medical insurance expenditure	Medical insurance fund expenditure volume	Rate variables	7.01367e+009 × time − 1.40673e+013	RMB/year
22	Total expenditure	Total expenditure of the maternity and health insurance pool	Rate variables	Maternity insurance fund expenditure + medical insurance expenditure	RMB/year
23	Total income	Total income of the maternity and health insurance pool	Rate variables	Maternity insurance fund income + medical insurance income	RMB/year
24	Total incentive payments	Total fertility incentive payments	Auxiliary variables	Average incentive payments × second child comprehensive (or all newborns)	RMB
25	Average incentive payments	Average incentive payment on fertility	Auxiliary variables	Assigning values based on different scenarios	RMB

Source: Jiangsu Province Statistical Yearbook and the National Bureau of Statistics of China and system dynamics simulation results

INTEG means integration, TFC means the average maternity incentive payments

## Results

### Goodness-of-fit tests

The historical data of the maternity insurance fund in Jiangsu Province were compared with the simulated data of the system dynamics model for the sustainable development of the future maternity insurance fund after the merger. The results show that the errors of all the tested variables are within 8%, with most of them within 4%. This indicates that the simulation of the system dynamics model of the future sustainable development of the maternity insurance fund after the merger is valid, and the pattern of changes in the simulation parameters is representative of the development trend of the variables in real life, which can be used for the next step of future scenario analysis and speculation. Table 2 shows the consistency test of the fund income and expenditure of the system dynamics model for the future sustainable development of the maternity insurance fund after the merger.

**Table 2:** Historical consistency test of the system dynamics model for the sustainability of the future maternity insurance fund after the merger of the two insurances

Year	Actual income of the maternity insurance fund (billions)	Simulated run-value	Relative error	Actual expenditure of the maternity insurance fund (billions)	Simulated run-value	Relative error
2011	25.8	25.65	−0.005	15.82	16.59	0.04
2012	32	31.54	−0.01	22.22	21.64	−0.02
2013	36.3	37.03	0.02	26.94	26.57	−0.01
2014	40.04	43.01	0.07	34.18	31.14	−0.08
2015	34.81	34.78	−0.0008	43.02	44.82	0.04

Year	Actual income of the medical insurance fund (billions)	Simulated run-value	Relative error	Actual expenditure of the medical insurance fund (billions)	Simulated run-value	Relative error
2011	421.94	466.09	0.09	338.01	372.44	0.09
2012	531.6	545.08	0.026	418.74	442.58	0.05
2013	611.1	624.06	0.02	496.12	512.72	0.03
2014	698.6	703.04	0.007	586.6	582.85	−0.006
2015	783.16	782.03	−0.001	666.98	652.99	−0.02

Source: Jiangsu Province Statistical Yearbook and the National Bureau of Statistics of China and system dynamics simulation results

### Simulation of the comprehensive two-child policy following the implementation of the merger of maternity and health insurance

**Fig. 3:**
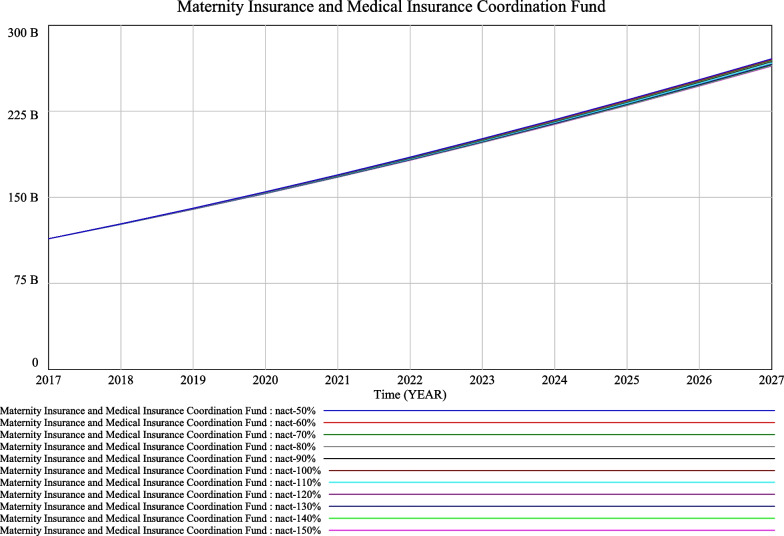
Simulation of the comprehensive two-child policy to the consolidated fund after the merger of the two insurance policies

Assume that the contribution rate remains unchanged at the current policy rate of 0.005 and that $({\text{nact}})_{t}$, the annual number of new children resulting from the comprehensive two-child policy, varies by 10% of 10 000 (100% $({\text{nact}})_{t}$ = 10 000). Assume the future change of the $({\text{nact}})_{t}$ number will be between 50% (lower limit value, $({\text{nact}})_{t}$ = 5 000) and 150% (upper limit value, $({\text{nact}})_{t}$ = 15 000). The trend in the fund balance is shown in Fig. 3.

Figure 3 shows that, regardless of the effect of a comprehensive two-child policy, the cumulative balance of the maternity insurance and health insurance pools would not experience a cumulative deficit and would continue to expand for at least the 10 years of the simulation. The trend of a steadily increasing fund balance will not change. This is because Jiangsu’s urban employees’ medical insurance fund has a large stock of funds and a high accumulated balance.

From Fig. 3 it can be concluded that when $({\text{nact}})_{t}$ is varied by 10% of 10 000 people, it has little impact on the future trend of the change in the cumulative balance of the maternity and health insurance pool after the system change is implemented, for all outcomes within a range of 50–150%. In order to explore the limit threshold of $({\text{nact}})_{t}$ relative to the future sustainable operation of the maternity and health insurance pool, the next simulation will expand the hypothetical value of $({\text{nact}})_{t}$ and compare its results with the simulation results of the actual conforming $({\text{nact}})_{t}$ value. The maximum tolerance of $({\text{nact}})_{t}$ under the theoretical assumptions of future system changes was used to avoid the real-world problem of deficits due to an increase in the number of newborns, and to prevent the problem from occurring in the future.

**Fig. 4:**
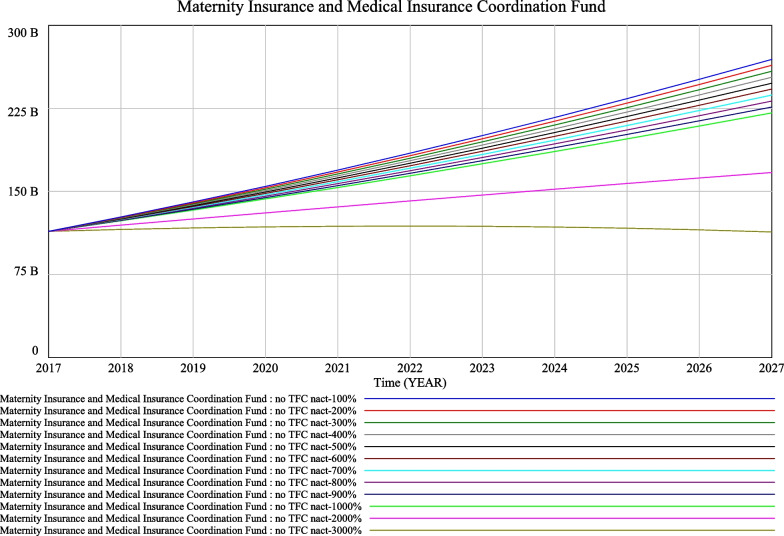
Simulation of the sustainable operation of the maternity insurance fund after future institutional changes under a comprehensive two-child policy

Assuming that the rate of contribution to the maternity insurance system remains unchanged at the current policy of 0.005 and that the annual number of new $({\text{nact}})_{t}$ births resulting from the comprehensive two-child policy changes by 100% of 10 000, running all results from 100 to 1 000%, two special $({\text{nact}})_{t}$ assignments are set up to explore the limit thresholds, namely 200 000 for 2 000% and 300 000 for 3 000%. The trend in the change in the cumulative balance of the maternity and health insurance funds after the future system change is shown in Fig. 4.

Figure 4 shows that when $({\text{nact}})_{t}$ is varied by 100% of 10 000 people, running all results from 100 to 1 000%, the overall trend in the accumulated balance of the maternity and health insurance funds remains constant in the future. However, as $({\text{nact}})_{t}$ increases significantly, the accumulated fund balance decreases and breaks even after $({\text{nact}})_{t}$ exceeds 300 000 persons, moving sharply into deficit thereafter. Therefore, the limit of the two insurance funds for $({\text{nact}})_{t}$ in Jiangsu Province after the future system change is around 300 000 persons. This serves as an early warning and also demonstrates that the implementation of the combined insurance fund had significantly improved the risk resistance and sustainability of the maternity insurance fund.

### Simulation of the incentive payment policy for second child only after the implementation of the merger of maternity and health insurance

As China does not yet have a comprehensive and systematic incentive policy, this study selects a convenient and quantifiable indicator of fertility incentives as the fertility incentive policy to be studied, and as China does not yet have a clear system of fertility incentives, the study will conservatively establish different incentive brackets based on the incentive payment standards of different countries around the world in order to conduct predictive simulations [6–11].

It is assumed that the maternity insurance contribution rate remains unchanged at 0.005 for the current policy, and that the number of new children per year $({\text{nact}})_{t}$ resulting from the comprehensive two-child policy is 10 000. The policy of giving people incentives to have children is assumed to be implemented from 2020, with scope for second children only. It means the families eligible for the maternity incentive payments must meet the condition that the birth of the second child in the family occurs after the implementation of the comprehensive two-child policy. As a result, the number of people receiving maternity incentive payments is very small at around 10 000 per year. We simulated the effect of different average incentive amounts on fund balances. The minimum amount of average maternity incentive payments is RMB 10 000, and each increment is RMB 10 000.

**Fig. 5:**
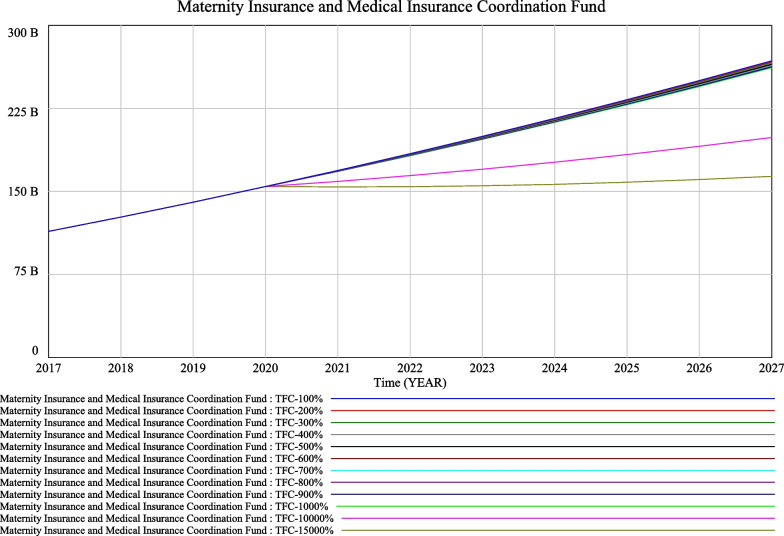
Simulation of the policy on fertility incentive payments for second child only

It can be seen from Fig. 5 that if the average maternity incentive payments are between RMB 10 000 (TFC-100%) and RMB 100 000 (TFC-1 000%), the impact on the sustainable operation of the maternity and health insurance pools over a few years is not significant and will not change the gradual increase in the accumulated balance. However, in the long term, as the maternity incentive payment increases, the accumulated balance of the maternity and health insurance funds will be depleted at an accelerated rate. The theoretical threshold for average maternity incentive payments has been calculated to be around RMB 1 500 000 (TFC-15 000%). The amount is so high because there are so few beneficiaries, excluding families who have just had their first child, and huge amounts of health-insurance income will be used as a fertility incentive.

Above this value, the maternity insurance and health insurance funds are rapidly depleted. However, as health insurance is a social insurance system established to compensate employees for financial losses due to the risk of illness, it is not possible to establish extraordinarily high average maternity incentive payments from the maternity insurance and health insurance funds.

### Simulation of the incentive payment policy for all newborns after the implementation of the merger of maternity insurance and health insurance

It is assumed that the maternity insurance contribution rate remains unchanged at 0.005 for the current policy, and that the number of new children per year $({\text{nact}})_{t}$ resulting from the comprehensive two-child policy is 10 000. The policy of giving people incentives to have children is assumed to be implemented from 2020, with the scope being all newborns. We simulated the effect of different average incentive amounts on fund balances. The minimum amount of average maternity incentive payments is RMB 10 000 (all child TFC-100%), and each increment is RMB 10 000.

**Fig. 6:**
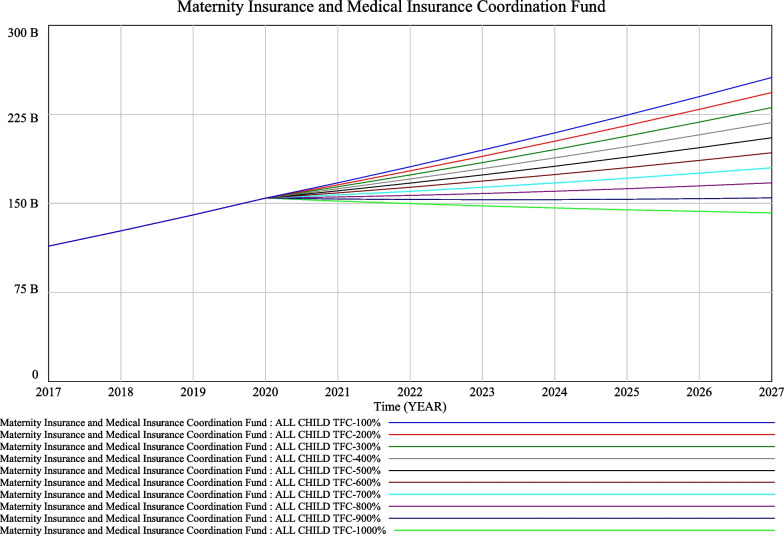
Simulation of the incentive payment policy for all newborns

As seen from Fig. 6, assuming that entitlement to the maternity incentive policy is extended to all newborns as insured persons, the impact on the sustainable operation of the insurance pools would be significant. At approximately RMB 90 000 above acceptable levels, the maternity and health insurance pools would enter a current deficit, rapidly depleting the accumulated balance of the fund. Given the fundamental role of health insurance, it would be prudent to set a level of maternity insurance incentives for all newborns; otherwise, it would not be conducive to the sustainability of the maternity insurance and health insurance funds.

## Discussion

The purpose of this paper was to explore the latest reform of China’s maternity insurance system and the implications for future incentive-based population policies. As a result of factors such as the effect of the comprehensive two-child policy, it is difficult to ensure the sustainability of the maternity insurance fund by relying on the original fund size and operation of the maternity insurance system alone. Even if sustainability could be achieved in theory, it would require significant adjustments to indicators such as contribution rates [2, 4]. However, because of the COVID-19 global pandemic and resulting poor economic conditions [31, 32], it is difficult to make changes to the original maternity insurance system and adjust the values of contribution rates to the theoretical values in the simulation results. The difficulty here is not that the government cannot make the adjustment, but that most enterprises and institutions are under great pressure in the current economic situation, making it more difficult to implement the adjustment. It would be even more arduous if there were to be a maternity incentive policy in the future. Therefore, if the maternity insurance fund is to remain sustainable, a reform approach that is more in line with the current situation is required. The best way to improve the fund’s ability to support a comprehensive two-child policy and a maternity incentive policy is to improve the risk resistance and sustainability of the maternity insurance fund [33, 34] and avoid putting too much pressure on enterprises and institutions.

The results indicate that a reasonable way to reform the maternity insurance system is to merge the two insurance policies in the future, as verified by simulation calculations. From an objective point of view, the accumulated balance of China’s urban employees’ medical insurance fund is 51 times that of the maternity insurance fund, and the combination of the two insurance systems will help to bring the deficit-ridden maternity insurance fund out of its predicament and achieve sustainable development. In reality, with 12 cities in China already piloting the scheme, the combination of health insurance and maternity insurance is the next step in the evolution of the maternity insurance system. The article also finds through simulation that the limit value of the annual number of new children resulting from the comprehensive two-child policy for the future combined fund in Jiangsu Province is around 300 000 people. Although this scenario only exists theoretically, and the insurance properties and roles of medical insurance are already covered by maternity insurance in this case, it also serves as an early warning. The results also demonstrate that the implementation of the merger of the two insurance policies has significantly increased the risk resilience and sustainability of the maternity insurance fund. This is in line with the results from previous studies which argue that fragmentation in the health insurance system may lead to inequity in financial access to and utilization of healthcare services. One possible option to overcome this challenge is merging of the existing health insurance funds [34, 35].

The significant impact of economic incentives on fertility promotion is now internationally recognized [6–11, 36]. With regard to the scenario of a maternity incentive policy, the paper shows that, based on simulation results, when the maternity incentive policy starts in 2020 for two children only, the trend of a gradual increase in the cumulative balance of the maternity insurance and health insurance pools will not change even if the maternity incentive increases from RMB 10 000 to RMB 100 000, due to the relatively small number of recipients. The simulation results are relatively approximate, as shown in Fig. 3. The impact of each RMB 10 000 increase on the maternity insurance and health insurance funds is very large when the maternity incentive policy is introduced for all newborns in 2020, with a regular fan-shaped scattering effect, as seen in Fig. 4. Only when the maternity incentive is assumed to be RMB 10 000 is the trend of increasing cumulative balances more or less the same as before. It is therefore recommended that when the scope of the fertility incentive policy is for two children only, an average amount above RMB 10 000 can be set. Caution is advised in setting the amount above RMB 10 000 when the scope of the fertility incentive policy is for all newborns.

The greater the amount of fertility incentive set in the implementation of the above responses, the more effective the policy incentive will be, but at the same time the more difficult it will be to implement. Setting a reasonable amount of fertility incentive is the only way to maintain the sustainability of the fund while playing a role in promoting fertility intentions to a certain extent. In the long term, a system of incentives for childbirth should be built from education policy, house price regulation, tax relief and childcare services [37–39].

This study has some limitations. Firstly, it was carried out in the single case of China’s maternity insurance system, and some concepts may need to be analysed on a case-by-case basis when being extended to other countries. Secondly, in reality, the incentive payments could also come from national coffers, but due to the global impact of infectious pneumonia, we are concerned that this may place an additional burden on national coffers. Therefore, we only simulated the situation where the incentive payments come from the expenditure of maternity insurance funds.

## Conclusion

This study analyses the factors influencing the sustainability of the maternity insurance fund through qualitative research. A system dynamics model for the future merger of the two insurance policies was constructed based on the reform trend of China’s maternity insurance. Simulations of the comprehensive two-child policy and the fertility incentive policy in Jiangsu Province were carried out. Our research not only highlights the significance of improving the resilience of maternity insurance by combining maternity insurance and health insurance funds, but also suggests a way to economically incentivize beneficiaries to have children so as to mitigate the decline in China’s birth rate and cope with the crisis of an ageing population.

## Data Availability

Please contact the corresponding author for data requests.

## Abbreviations

UWMIF: Urban workers’ medical insurance fund
URMIF: Urban residents’ medical insurance fund
PIF: Pension insurance fund
NRCF: New rural cooperative fund
WIIF: Work injury insurance fund
nact: The number of new second children with maternity insurance
TFC: The average maternity incentive payments
